# An Electrochemical Sensor Based on Nanostructured Hollandite-type Manganese Oxide for Detection of Potassium Ions

**DOI:** 10.3390/s90906613

**Published:** 2009-08-24

**Authors:** Alex S. Lima, Nerilso Bocchi, Homero M. Gomes, Marcos F. S. Teixeira

**Affiliations:** 1 Department of Physics, Chemistry and Biology, Faculty of Science and Technology, University of State of Sao Paulo (UNESP), Rua Roberto Simonsen 305, CEP 19060-900 Presidente Prudente - SP, Brazil; E-Mails: silvalima_alex@yahoo.com.br; homago@fct.unesp.br; 2 Departamento de Química – Universidade Federal de São Carlos, São Carlos – SP, Brazil; E-Mail: bocchi@dq.ufscar.br

**Keywords:** hollandite-type manganese oxide, potassium ion, insertion electrochemical

## Abstract

The participation of cations in redox reactions of manganese oxides provides an opportunity for development of chemical sensors for non-electroactive ions. A sensor based on a nanostructured hollandite-type manganese oxide was investigated for voltammetric detection of potassium ions. The detection is based on the measurement of anodic current generated by oxidation of Mn(III) to Mn(IV) at the surface of the electrode and the subsequent extraction of the potassium ions into the hollandite structure. In this work, an amperometric procedure at an operating potential of 0.80 V (*versus* SCE) is exploited for amperometric monitoring. The current signals are linearly proportional to potassium ion concentration in the range 4.97 × 10^−5^ to 9.05 × 10^−4^ mol L^−1^, with a correlation coefficient of 0.9997.

## 1. Introduction

Potassium monitoring in serum, urine, and foods is very important in the clinical and medical fields, being one of the most important routine analysis performed in a clinical laboratory [1]. From the potassium determination, medical information concerning physical conditions of the patients can be obtained in the cases where renal diseases, hypopotassemia, alkalosis, cirrhosis of liver, diuretic drugs, etc. are suspected. On the other hand, when the potassium concentration in human serum becomes higher than 9 mmol L^−1^, the heart often stops [2]. Hence, accurate, easy and rapid sensing of potassium ions is very important.

The usual methods for the determination of potassium ion involve flame photometry [3], colorimetric methods [4,5], and ion-selective electrodes [6,7]. The development of chemical sensors for non-electroactive species has been proposed based on the participation of non-electroactive cations in redox reactions of metal hexacyanoferrates [8]. Another compound with the ability of accommodate non-electroactive cations and promote the electroactivity in function of the insertion cation is manganese oxide.

Manganese oxides represent a large class of materials that have layered and tunneled nanostructures consisting of edge-shared MnO_6_ octahedral units. They have attracted considerable interest due to their broad potential applications in heterogeneous catalysis, chemical sensing, and rechargeable battery technology [9,10]. In our laboratories, we are also interested in developing highly sensitive and selective methods for the determination of non-electroactive cations using electrodes modified with different allotropic forms of manganese oxide [11–15]. The manganese oxides have distinct advantages over other materials for various applications due to the existence of mixed manganese valencies (mainly, 4+, 3+, or 2+) [16].

Hollandite-type (or cryptomelane) manganese oxides are one-dimensional (2 × 2) tunnel (0.46 nm) nanostructures formed by interlinking the edge-shared MnO_6_ octahedral units. Manganese in the structure is mainly present as Mn(IV) and Mn(III), and cations (K^+^ and H^+^) with a small amount of water occupy the tunnel or lay between the layers to provide charge balance. According to Feng *et al*. [17], the K^+^ extraction and metal ion adsorption reactions are topotactic, preserving the hollandite structure. The following redox mechanism was proposed for the potassium ions extraction reaction from {K_2_}[Mn^III^Mn^IV^]O_16_ hollandite [18]:

(1) $$8\left\{K_{2}\right\}{\left[{\text{Mn}}^{\text{III}}\right]}_{2}{\left[{\text{Mn}}^{\text{IV}}\right]}_{6}O_{16(s)}+{{32H}^{+}}_{\left(\text{aq}\right)}\to 7\{\;\}{\left[{\text{Mn}}^{\text{IV}}\right]}_{8}O_{16(s)}+16{K^{+}}_{\left(\text{aq}\right)}+{{8\text{Mn}}^{2+}}_{\left(\text{aq}\right)}+{16H}_{2}O_{\left(1\right)}$$

where { } and [ ] are the tunnel and octahedral sites, respectively. The extraction of potassium ion accompanies the disproportion of Mn(III) to Mn(IV) and Mn(II).

In the present work, the preparation, properties and application of an electrochemical sensor based on hollandite-type MnO_2_ for the amperometric determination of potassium ions was studied. The influence of various parameters, such as electrode composition, pH electrolyte, and interference of several ions on the voltammetric profiles of the proposed electrode is also presented. The amperometric measurements realized are based on the topotactic electrochemical reaction of insertion/extraction of the non-electroactive cation in the hollandite structure.

## 2. Experimental Section

### 2.1. Reagents and Solutions

All solutions were prepared using a Millipore Milli-Q water. All chemicals were analytical reagent grade, used without further purification. The supporting electrolyte used for all experiments was a 0.1 mol L^−1^ tris(hydroxymethyl)aminomethane buffer solution (TRIS, pH = 8.3). A 0.01 mol L^−1^ potassium ions solution was prepared daily by dissolving potassium chloride (Merck) in 100 mL of TRIS buffer. Graphite powder (1–2 μm particle size from Aldrich) and mineral oil (Aldrich) of high purity were used for the preparation of the carbon pastes.

### 2.2. Apparatus

The powder X-ray diffractograms (XRD) was obtained using a Siemens D-5000 automated diffractometer with Cu K_α1_ radiation from 5° to 75° at a scanning speed 0.02°/min. All voltammetric measurements were carried out in a 25 mL thermostatic glass cell at 25°C containing three electrodes: carbon paste electrode modified with holladite-type manganese oxide (sensor) as working electrode (0.196 cm^2^ areas), a saturated calomel electrode (SCE) as the reference, and a platinum auxiliary electrode. The electrochemical behavior and activity of the sensor were evaluated in the potential range from −0.25 V to +1.0 V *vs*. SCE at a scan rate of 50 mV s^−1^ in a 0.5 mol L^−1^ tris(hydroxymethyl) aminomethane (TRIS) buffer. Chronoamperometrics measurements were realized at an operational potential of +0.80 V *vs*. SCE. These measurements were performed with an μ-Autolab type III (Eco Chimie) controlled by a computer.

### 2.3. Preparation of Hollandite-Type MnO_2_

Hollandite-type manganese(IV) oxide was prepared using a sol-gel route according to Ching *et al.* [25] and a redox precipitation route according to DeGuzman *et al.* [26].

*Sol-gel route*: The cryptomelane as prepared a sample of solid fumaric acid was added to a stirred solution containing KMnO_4_ in a 1:3 mole ratio generates a brown sol. The gel allows it to be washed with water before drying, and calcination of the resulting xerogel at 450 °C for 2 h.

*Redox precipitation*: 100 mL of a solution of KMnO4 0.37 mol L^−1^ was added to 30 mL of a solution of MnSO_4_.H_2_O 1.37 mol L^−1^ and 3 mL concentrated HNO_3_. The solution was refluxed at 100 °C for 24 h, and the product was filtered, washed, and dried at 120 °C.

For conversion to hollandite-type manganese oxide (α-MnO_2_), a cryptomelane sample was treated in an aqueous 1M nitric acid solution for 2 days. The sample was filtered, washed with deionized water, and air-dried at 70 °C.

### 2.4. Construction of the Carbon-Paste Electrodes

Carbon-paste electrodes modified (CPEM) with hollandite-type MnO_2_ was prepared by carefully mixing the dispersed graphite powder with manganese oxide at varying ratios. Exactly 1 g of this mixture was subsequently added to 0.200 g of mineral oil (20% m/m) and mixed in a 50 mL beaker containing 20 mL of hexane. The final paste was obtained with the solvent evaporation. The carbon-paste electrode was finally obtained packing the paste into a plastic tube and arranged with a copper wire serving as an external electric contact. The electrode surface was smoothed on a weighing paper. When necessary, a new electrode surface as obtained by removing about 2–3 mm from surface of electrode material, adding freshly made oxides/carbon-paste mixture, and polishing it.

## 3. Results and Discussion

### 3.1. Characterization of Hollandite-type Manganese oxide

**Figure 1.**
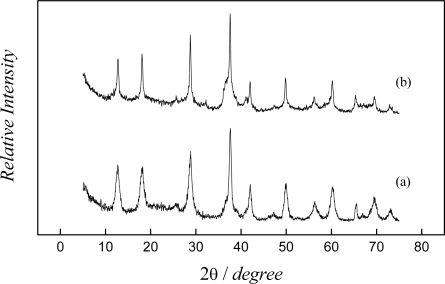
Powder X-ray diffraction pattern of hollandite-type manganese oxide prepared by: (a) redox precipitation, (b) sol-gel route.

The corresponding XRD patterns were obtained (Figure 1) to confirm the conversion of the synthesized materials. The intense and sharp diffraction peaks at 2θ = 12.6°, 17.9°, 28.7°, 37.5°, 41.9°, 49.9°, and 60.1° can be attributed to the crystalline phase of hollandite (JCPDS File No. 44-0141), indicating that the samples maintained the structure after acid treatment.

The width peaks in the XRD pattern of the sample synthesized by sol-gel suggests that the oxide powder shows higher degree of crystallinity when compared to the sample synthesized by redox precipitation. This can be due to a variation of the potassium content [18] in each domain during the synthesis of the material (K/Mn atomic ratio = 0.19 for redox precipitation and K/Mn atomic ratio = 0.29 for sol-gel). The crystallite sizes estimated according to the Scherrer equation [19] were 23.9 nm and 17.0 nm for samples synthesized by sol-gel and redox, respectively. The resulting manganese oxide lattice formed may have more active sites when nucleation is slow and crystals are allowed to grow when compared to that of synthesis redox precipitation, in which crystals did not grow much.

### 3.2. Electrochemical Behavior of the Sensor

**Figure 2.**
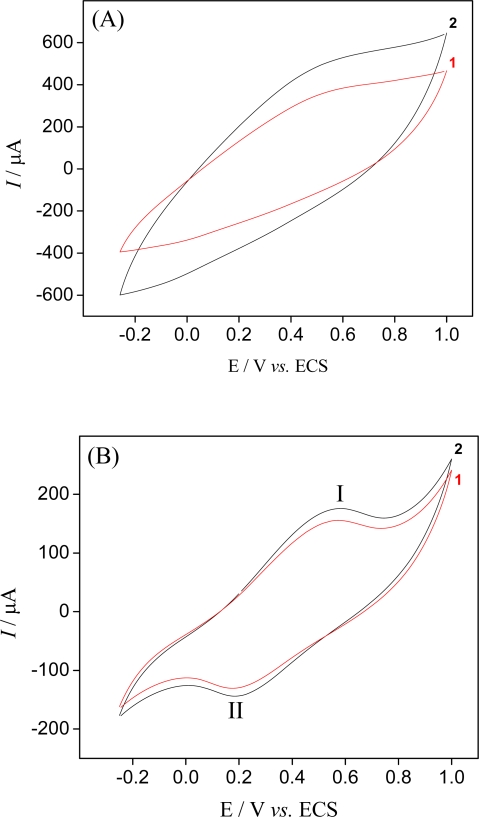
Cyclic voltammograms obtained at a scan of 50 mV s^−1^ for carbon paste modified with 20% (m/m) hollandite-type MnO_2_: (1 – red curve) in TRIS buffer solution (pH = 8); (2 – black curve) in TRIS buffer solution containing 4.8 × 10^−4^ mol L^−1^ of potassium ions. Hollandite manganese oxide synthesized by (A) redox precipitation; (B) sol-gel route.

The electrochemical and ion-sieve properties of the manganese oxides are frequently dependent on the synthetic process. The effect of the synthetic process on the electrochemical activity of the sensor based on hollandite-type manganese oxide was investigated by cyclic voltammetry. Figure 2 shows the cyclic voltammograms for samples synthesized by sol-gel and redox precipitation.

The carbon paste electrode modified with hollandite manganese oxide and synthesized by the sol-gel method showed a better electrochemical behaviour for potassium ions (see Figure 2B). This performance is related to the crystallinity degree and homogeneity of the oxide prepared. The particle shape and size of manganese oxide affect the way these particles are dispersed in carbon powder in sensor preparation. Larger particles favor a better dispersion where more of the manganese oxide is exposed and readily available for electrochemical activity. This surface may have more active redox sites as compared to that one of material synthesized by redox precipitation. The voltammetric profile of the sensor presented one anodic peak (peak I = +0.58 V *vs*. SCE) and another cathodic peak (peak II = +0.18 *vs*. SCE). This electrochemical activity is due to the extraction/insertion topotactic processes of the potassium ions from the hollandite structure [17]:

(2) $$\left\{K_{x}{□}_{2-x}\right\}{\left[{\text{Mn}}^{\text{III}}\right]}_{y}{\left[{\text{Mn}}^{\text{IV}}\right]}_{8-y}O_{16(s)}⇌\left\{{□}_{2}\right\}{\left[{\text{Mn}}^{\text{IV}}\right]}_{8}O_{16(s)}+{{\text{xK}}^{+}}_{\left(\text{aq}\right)}+{\text{xe}}^{-}$$

where { } denotes (2 × 2) tunnel sites, [ ] octahedral sites occupied by manganese, and ≤ vacant structural sites. The enhanced response to potassium ions occurs because the cathodic polarization of the sensor with hollandite-type manganese oxide is quite enough to reduce the manganese in the solid. Consequently, the potassium ions from the adjacent solution are able to diffuse through the hollandite structure to maintain the electroneutrality principle. In the absence of potassium ions (curve 1– Figure 2B), the voltammetric profile of the sensor shows a redox peak. Supposedly, that behavior can be related to vestiges of potassium ions in the hollandite-type manganese oxide that may have occurred during its preparation. The acid-treatment extracts 48% of K^+^ ions from the metal ion inserted sample. This suggests that K^+^ with a large ionic radius is tightly fixed on the (2 × 2) tunnel sites. In the presence of potassium ions (curve 2–Figure 2B), increase voltammetric response was observed for the sensor, confirming that the electrochemical response is a function of the insertion reaction of potassium ions in the hollandite structure.

The sensor composition was also investigated to check if the electrochemical response was limited by the electronic conductivity of the composite electrode. The amount of binder was kept constant while varying the amount of hollandite-type manganese oxide and graphite. The amount of manganese oxide in the carbon paste had a significant influence on the voltammetric response. The peak currents increase with amount of hollandite-type manganese oxide up 20 % (m/m). For amounts of manganese oxide higher than 25% (m/m) the anodic peak current decreased significantly. This occurs due to a decreasing of the graphite content in the paste and consequently the reduction of conductive area at the electrode surface. The best carbon-paste composition was found for an electrode composition of 20% (m/m) hollandite-type manganese oxide synthesized by sol-gel, 65% (m/m) graphite and 15% (m/m) mineral oil was used in further studies.

The variation of the peak currents and the potential peak separation (Δ*E*_p_ = *E*_pa_ − *E*_pc_) of the sensor based on hollandite-type managanese oxide in solution containing 4.8 × 10^−4^ mol L^−1^ potassium ions with scan rate (1–100 mV s^−1^) was studied. The Δ*E*_p_ increase with variation of scan rate. These results allow to conclude that the charge transfer reaction of Mn^(IV)^/Mn^(III)^ is controlled by diffusion of potassium ions in the solid phase. Hence, the solid-state diffusion of K^+^ ions can be considered as the rate-determining step. The electrochemical insertion of potassium ions into the structure in the hollandite-type manganese oxide consists of three processes that are: solution mass transport, dehydration and transfer at the solid surface, and solid-state diffusion [18].

Linear relations were obtained when plotting the peak currents versus the square root of the scan rate revealing a diffusion controlled rate reaction. The following linear relationships were obtained:

(3) $$I_{\text{pa}}\;(\mu A)=-26.7+34.8\;v^{½}\;\left({\text{mV}}^{1/2}\;s^{-1/2}\right)\;r=0.9991$$

(4) $$I_{\text{pc}}\;(\mu A)=51.1+33.5\;v^{½}\;\left({\text{mV}}^{1/2}\;s^{-1/2}\right)\;r=0.9999$$

**Figure 3.**
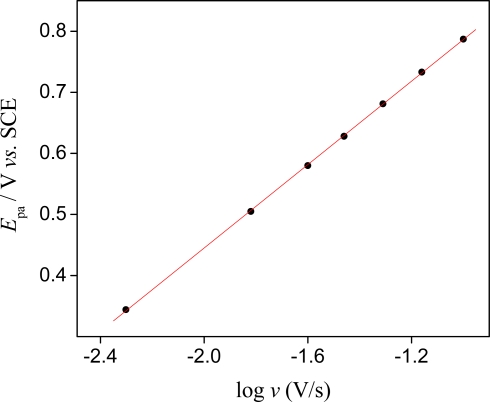
Dependence of E_pa_ with log(*ν*) for the sensor in Tris buffer solution (pH = 8) containing 4.8 × 10^−4^ mol L^−1^ potassium ions.

The apparent electrochemical rate constant *k*_e_ and the electron-transfer coefficient *α*_anodic_ were calculated for the sensor according to the method described by Larivon [20,21]. It has been shown by Laviron that for a surface redox couple, *α*_anodic_ and *k*_e_ can be determined from the variation of *E*_pa_ with scan rate. Figure 3 presents the plot of *E*_pa_ (V) *versus* log *ν* (V s^−1^) of the sensor in Tris buffer solution (pH = 8) containing 4.8 × 10^−4^ mol L^−1^ potassium ions.

The *E*_p_−log *ν* plots gave one straight line with slopes of 2.303*RT*/(1−*α*_anodic_)*nF* for the anodic branch, where *R* is the gas constant, *T* the absolute temperature, *F* the Faraday constant and *n* number of electrons involved in the redox couple. Considering that the number of electrons involved in the redox process is 1, the calculated value for the coefficient *α*_anodic_ was 0.83. These results suggest the redox process tends towards an irreversible system. The apparent electrochemical rate constant can then be determined by applying the equation *k*_e_ = 2.303*α*_anodic_*nFν*_o_/*RT*, in which the value of scan rate (*ν*_o_) is determined by extrapolation of the linear branch at higher scan rates and its intersection with the constant peak potential, represented by the peak of the voltammogram at the lower scan rate. The observed value was *k*_e_ = 0.4 s^−1^.

The influence of the pH on the electrochemical response of the sensor was studied over a pH range between 4.5–9 controlled with TRIS buffer in the absence and in the presence of potassium ions of 4.8 × 10^−4^ mol L^−1^. The cyclic voltammograms of the sensor based on hollandite-type MnO_2_ were performed at scan rate of 50 mV s^−1^ in different pHs.

**Figure 4.**
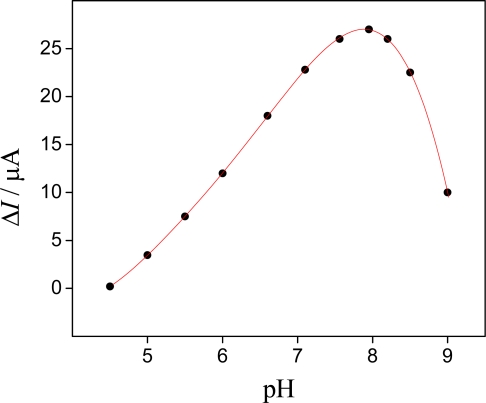
pH dependence of the voltammetric response of the sensor in a TRIS buffer solution of different pHs in presence of 4.8 × 10^−4^ mol L^−1^ potassium ions. Scan rate = 50 mV s^−1^. The anodic peak current (Δ*I*_pa_/*μ*A) was obtained by the difference of the currents in the presence and absence of potassium ions.

The obtained result indicated that the current value of anodic peak is strongly influenced by the pH and reached a maximum value at pH 8 (see Figure 4). According to Feng *et al*. [17], extraction of potassium ions in structure and surface disproportionation reaction of the manganese oxide (2Mn^3+^_(s)_ → Mn^4+^_(s)_ + Mn^2+^_(aq)_) in the presence of cation ions occur at acid pH. With this disproportionation reaction, the amount of manganese oxide in the electrode surface also decreases, leading to smaller peak currents. For pH 8.5 the value of peak current decreased considerably due to another surface formation of non-electroactive manganese oxide. Recently, Teixeira *et al.* [12,13] obtained similar behavior when studied the voltammetric properties of a carbon-paste electrode modified with spinel-type manganese oxide.

The dependence of anodic peak current of the sensor with the pre-concentration was also investigated. The voltammetric responses of the sensor were realized in a solution containing 4.8 × 10^−4^ mol L^−1^ of potassium ions after pre-concentration at −0.25 V *vs*. SCE for different times. The difference of the anodic peak currents in the presence and absence of potassium ions increases with the increasing of the pre-concentration time between 0 and 100 s. It has become nearly constant due to the surface saturation of the oxide with potassium ion. Based on this experiment, it is demonstrated the ability of the hollandite to accommodate non-electroactive cation and promote the electroactivity in function of the insertion potassium is the manganese oxide.

### 3.3. Analytical Performance of the Sensor

The influence of alkaline (Na^+^, K^+^, Rb^+^ and Cs^+^) and earth alkaline (Mg^2+^, Ca^2+^, Sr^2+^, Ba^2+^) ions on the response of the sensor has been studied. For this study, the current values of the sensor were obtained by injection of sample volumes containing alkaline and alkaline-earth ions in the absence and presence of potassium ions. Table 1 shows the relative current (%) calculated by the difference of the currents in the presence and absence of the cation in study.

A characteristic feature is that the selectivity sequences relate to the pore size of the manganese oxide. The selectivity sequences are Cs^+^ < Rb^+^ < Na^+^ ≤ Li^+^ < NH_4_^+^ < K^+^ and Mg^2+^ < Ca^2+^ < Sr^2+^ < Ba^2+^ < K^+^ for sensor based on holladite-type manganese oxide. The hollandite-type MnO_2_ ion-sieves show a large adsorptive for potassium ion at low pH range [18,22]. However, at high pH range, the adsorptive capacity increases with a decrease of ionic radius, except for alkaline-earth ions. This suggests that the insertion reaction is subject to the steric interaction between the cation ions in the tunnel and it becomes predominant. When electrochemical measurements were recorded in solution containing potassium ions, it was not observed a significant increase in the relative current indicating that the insertion reaction occurs preferably with potassium ions. This behavior is related to cavity size of the MnO_2_, dehydration energy and especially to ionic mobility of the metallic ion in aqueous solution. Potassium ions are expected to be more mobile than lithium, sodium, all alkaline-earth ions, and might therefore enhance the rate of charge transfers, evidenced by decreasing the influence of these cations in the relative currents.

**Figure 5.**
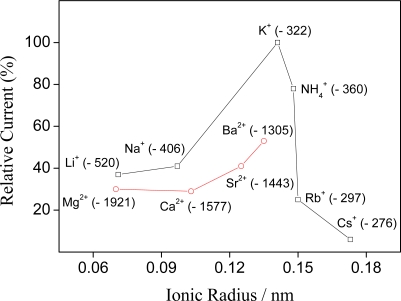
Plot of relative current of the sensor *versus* ionic radius of alkaline and alkaline-earth ions. The values in parentheses represent the dehydration energy (23) for each cation study.

The relative currents of the sensor observed upon addition of alkali and alkaline earth metal ions into a 4.8 × 10^−4^ mol L^−1^ potassium ion background electrolyte solution were plotted as a function of ionic radius of the cations in Figure 5 (with respective enthalpies of dehydration). As it is seen, there are obviously size selectivities to the ions with an ionic radius around 0.141 nm. Ammonium ion caused a serious interference on the electrochemical response of the sensor. As the ionic radius of ammonium ion (0.148 nm) is close to that of potassium ion (0.141 nm), NH_4_^+^ can be inserted into the tunnel structure of hollandite. The weaker responses to divalent cations compared to monovalent ions having similar ionic radius may be due to their higher energies of dehydration which is needed for uptake of cations into the solid phase from the aqueous solution. Hydration of an ion depends on the electrostatic attraction of water molecules to that ion. Because attraction of water molecules around an ion depends on that ion's density of charge, smaller ions (and thus ions of greater ionic potential) attract more water molecules. The result is the inverse relationship between non-hydrated radius and hydrated radius. The relatively small amount of Li^+^ and Na^+^ loading suggests that lithium and sodium ions are inserted into the tunnel in a partially hydrated form.

**Figure 6.**
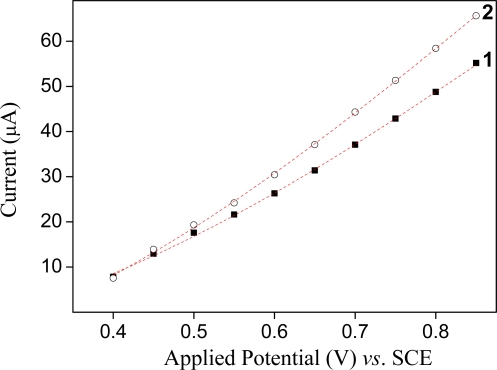
Influence of the applied potential on the anodic current of the sensor. (1) background current. (2) peak height of the current response.

The optimal potential of the working electrode had to be found to achieve the most sensitive determination of the potassium ion. Thus, we studied the dependence of current responses on potential in the range from 0.4 to 0.9 V *vs*. SCE in the absence and presence of the potassium ion under continuous stirring at 300 rpm. The resulting hydrodynamic voltammograms are shown in Figure 6. The height of current responses of the potassium increased with increasing potential. However, the background signal (absence of the potassium ions) was too high with the increase of applied potential. An operating potential of +0.80 V vs. SCE was selected for all further studies due to the good reproducibility and stability of the sensor.

In order to obtain an analytical curve for the developed sensor, chronoamperometric studies for potassium determination were carried out at different concentrations in 0.1 mol L^−1^ TRIS (pH 8.0) under continuous stirring at 300 rpm. The measurements were realized with applied at potential of −0.25 V *vs*. SCE by 30 s and then was at potential level of 0.80 V. The useful net current signals recorded at 60 seconds.

**Figure 7.**
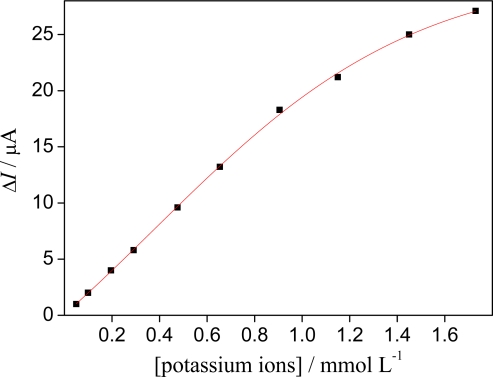
Calibration curve for potassium ion using sensor based on hollandite-type manganese oxide. Potential step: −0.25 V to 0.80 V *vs*. SCE. Stirring rate: 300 rpm

The proposed sensor showed a linear response range from 4.97 × 10^−5^ up to 9.05 × 10^−4^ mol L^−1^ (Figure 7), which can be expressed according to the following equation Δ*I* (μA) = 0.007 + 20.2 [K^+^] (mmol L^−1^) with a correlation coefficient of 0.9997 (n = 7). At higher concentrations (> 9.05 × 10^−4^ mol L^−1^), deviation from linearity occurs. The detection limit calculated according to the recommendations of IUPAC [24] were 1.61 × 10^−6^ mol L^−1^ of potassium ions, presenting good sensitivity of this sensor for potassium dosage.

## 4. Conclusions

Hollandite-type manganese oxide shows strong promise for potential application as an amperometric sensor for potassium ions. The mechanism of the sensor depends on the electrochemical activity of the manganese oxide with extraction/insertion topotactic processes of the potassium ions from the hollandite structure. With an operating potential of +0.80 V *versus* SCE, the current signals are linearly proportional to potassium ion concentration in the range 4.97 × 10^−5^ to 9.05 × 10^−4^ mol L^−1^ with a correlation coefficient of 0.9997. During successive cycles (100 cycles) in supporting electrolyte, the peak currents decreased by less 7%. The lifetime of the sensor was approximately seven months.

## Figures and Tables

**Table 1.** Interference dependence of the voltammetric responses of the sensor based on hollandite-type manganese oxide in TRIS buffer solution (pH = 8) containing 4.8 × 10^−4^ mol L^−1^ of alkaline and alkaline-earth metal ions in the absence and presence of potassium ions (4.7 × 10^−4^ mol L^−1^).

	**Absence of K^+^ ions**	**Presence of K^+^ ions**

**metallic cation**	***I*_relative_ (%)**	***I*_relative_ (%)**

K^+^	100	100
Li^+^	67	37
Na^+^	64	41
Rb^+^	53	25
Cs^+^	36	6
Mg^2+^	35	30
Ca^2+^	43	29
Sr^2+^	50	41
Ba^2+^	71	53
NH_4_^+^	86	78
